# The zinc contraceptive effect: targets, timing, and quantitative thresholds

**DOI:** 10.1242/bio.062546

**Published:** 2026-07-03

**Authors:** Kirsten Shankie-Williams, Margot Day, Samson Dowland

**Affiliations:** School of Medical Sciences, Faculty of Medicine and Health, The University of Sydney, Sydney, NSW 2006, Australia

**Keywords:** Contraception, Embryo, Zinc, Mechanism

## Abstract

There is a critical need for novel non-hormonal contraceptives. Zinc-based intrauterine devices (IUDs) demonstrate 100% efficacy in rats, yet the mechanism remains unclear. We investigated whether zinc targets sperm, embryos, or the endometrium. Rats with zinc, copper, or nylon IUDs were mated. All retrieved embryos were fertilised, indicating no primary spermicidal effect. However, embryos from rats with a zinc IUD failed to reach the blastocyst stage, unlike those from rats with a copper IUD. *In vitro* embryo culture confirmed that zinc significantly inhibited mouse embryo development in a dose-dependent manner, with 5 μg/ml zinc causing a 97% decrease in blastocyst formation, whilst copper caused a 61% decrease. Surviving zinc-treated embryos also had significantly reduced attachment to human endometrial cells, unlike copper-treated embryos. There was also no reduced attachment of untreated mouse embryos to zinc- and copper-treated endometrial cells. These findings suggest that zinc's primary contraceptive mechanism is the potent inhibition of pre-implantation embryo development.

## Teaser

**Summary:** The emerging non-hormonal zinc-based contraceptive has a first-of-its-kind target; rather than impacting the female reproductive system, zinc can inhibit development of the very-early embryo.

## INTRODUCTION

The only non-hormonal long-acting reversible contraception (LARC) is the copper intrauterine device (IUD). This device is an attractive option as it eliminates the possibility of experiencing systemic side effects caused by exogenous hormones, present in all other LARCs. However, the copper IUD causes increased menstrual bleeding and pain in 67% of women within the first year; thus, it is not advised for women with normal to heavy menstrual blood loss (Farr and Amatya, 1994; Hubacher et al., 2009). An alternative non-hormonal LARC has recently been proposed, employing the contraceptive effects of a highly biocompatible essential metal, zinc (Shankie-Williams et al., 2022). Within the uterine cavity of rats, zinc is able to provide 100% effective, long-term and reversible contraception with little morphological change to the endometrium, unlike endometrium exposed to copper (Shankie-Williams et al., 2022).

Zinc is relatively harmless to humans and is found at a mean serum concentration of 1000 μg/ml, considerably higher than that of copper, found at 0.1 μg/ml. The average concentration of copper in the uterine fluid (no IUD) is around 0.8 μg/ml, whilst for those using the copper IUD the concentration during the secretory and proliferative phase is 4.1 μg/ml and 3.9 μg/ml, respectively (Arancibia et al., 2003). Although, the concentration required for the contraceptive effect of copper is currently unknown (Plum et al., 2010), it can be assumed that the peak concentration of 19.1 μg/ml during menstruation is more than quadrupling the required concentration during the fertile period. At this level, copper is cytotoxic to around 75% of surrounding cells and considered to be a potential cause of the increased bleeding, often experienced by users (Farr and Amatya, 1994; Wu et al., 2012).

The mechanism of action (MOA) of the copper IUD has been retroactively studied since its initial clinical use in 1969, and multiple mechanisms have been suggested, including inhibition of sperm motility and acrosome reaction, the foreign body inflammatory response, inhibition of embryo development and inhibition of implantation via a further myriad of mechanisms (Carrascosa et al., 2018; Kesserü and Camacho-Ortega, 1972; Ortiz and Croxatto, 2007; Polidoro and Black, 1970; Roblero et al., 1996). Despite its prescription as a spermicide, the exact MOA remains unknown (Bunting et al., 2024).

While examining the contraceptive potential of zinc, we aimed to determine the target pathway, and whether it differed from copper. Zinc is transported and stored by the metallothionein protein to prevent oxidative stress from free metal ions (Babula et al., 2012). Metallothionein plays such a critical role in zinc homeostasis that it is transcribed from the maternal transcript from fertilisation (Andrews et al., 1991). At the early stages of embryo development, however, metallothionein expression is not yet metal responsive, and this is thought to give rise to a unique mechanism of zinc sensitivity in the two-cell embryo, wherein increased concentrations of zinc are lethal (Andrews et al., 1991; Vidal and Hidalgo, 1993). We hypothesised that a zinc-based IUD would produce an increased concentration of exogenous zinc ions in the environment surrounding the early embryo, preventing blastocyst development and pregnancy (Vidal and Hidalgo, 1993).

**Fig. 1.**
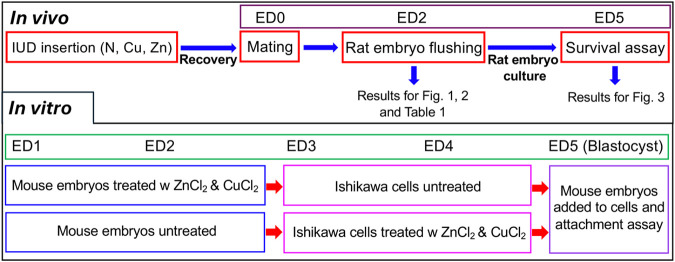
Flowchart of *in vivo* and *in vitro* exposure to zinc and subsequent rodent embryo survival.

The current study evaluated the hypothesised target of a zinc IUD *in vivo* and determined the minimum concentration of zinc required for contraceptive efficacy *in vitro* (Fig. 1). Concentrations between 1 μg/ml and 5 μg/ml were selected using an initial optimisation experiment. This narrowed down the range to capture the minimum concentration of zinc for significant contraceptive efficacy whilst including the smallest concentrations of copper found in the uterine fluid of copper IUD users before and after ovulation (Arancibia et al., 2003).

## RESULTS

### Copper and zinc IUDs do not prevent fertilisation

Pre- and post-fertilisation actions of the zinc and copper IUD were assessed by extracting embryos from rats mated with IUDs in place. On embryonic day (ED)2 all embryos collected from rats containing a copper IUD were fertilised, and all embryos from both the zinc IUD- and nylon IUD-treated rats were fertilised except one oocyte from each (Table 1). All embryos in contralateral control horns were also fertilised.

**Table 1.** Number of fertilised and unfertilised embryos collected from copper, zinc and nylon

	Cu IUD (*n*=5)	Cu Ctrl (*n*=5)	Zn IUD (*n*=5)	Zn Ctrl (*n*=5)	N IUD (*n*=3)	N Ctrl (*n*=3)
Fertilised	38	41	32	42	30	28
Unfertilised	0	0	1	0	1	0

Cu IUD, copper IUD; Cu Ctrl, contralateral control horn in copper IUD animal; Zn IUD, zinc IUD; Zn Ctrl, contralateral control horn in zinc IUD animal; N IUD, nylon IUD; N Ctrl, contralateral control horn in nylon IUD animal.

### The zinc IUD impacts early embryo health

**Fig. 2.**
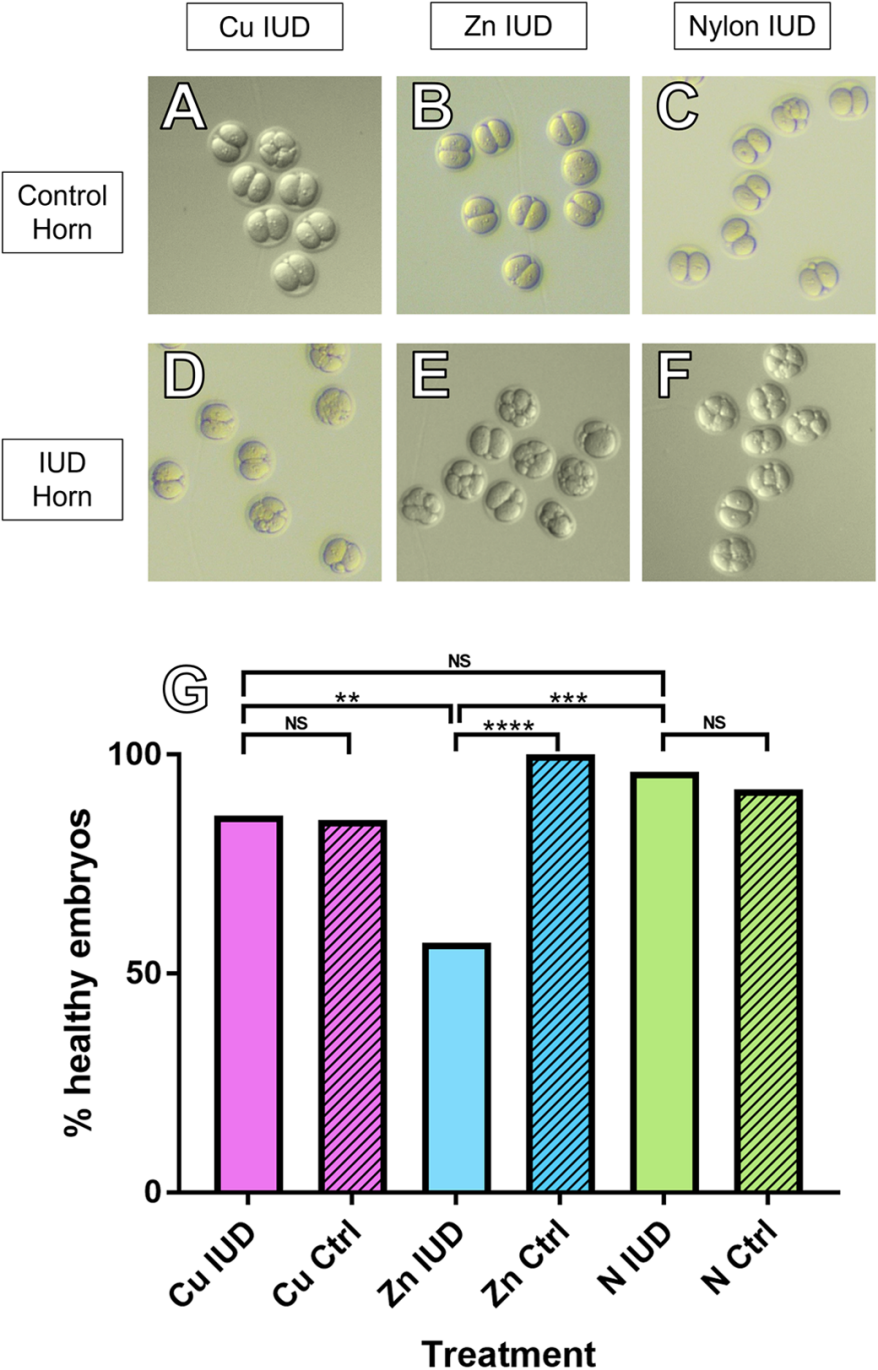
**Percentage of healthy embryos extracted from oviducts connected to IUD-treated and control uterine horns on ED2 (rat embryos at two- to four-cell stage).** (A-F) Representative images of Cu IUD (*n*=38), Cu Ctrl (*n*=37), Zn IUD (*n*=33), Zn Ctrl (*n*=41), N IUD (*n*=30) and N Ctrl (*n*=32) pooled from at least three experiments. (G) Chi-squared analysis with Fisher's exact test was employed, and Bonferroni adjusted *P*-values (*P*<0.008) were used to determine significance for specific comparisons. **P<0.008, ***P<0.001 and ****P<0.0001. Ctrl, control; N, Nylon; NS, not significant.

Embryo quality was determined by normal cleavage (i.e. even-sized blastomeres produced) and division to the appropriate stage on ED2 (Fig. 2A-C). Significantly fewer embryos from the zinc IUD-treated horns were healthy compared to those from the control horn (56% versus 100%; *P*<0.0001), the nylon IUD horn (56 versus 97%; *P*<0.0002) and copper IUD horn (56% versus 87%; *P*<0.0073) (Fig. 2). The majority of zinc IUD-treated embryos arrested at the two-cell stage. The copper and nylon IUDs did not significantly impact the development of embryos on ED2.

### Early exposure to the zinc IUD prevents blastocyst development

**Fig. 3.**
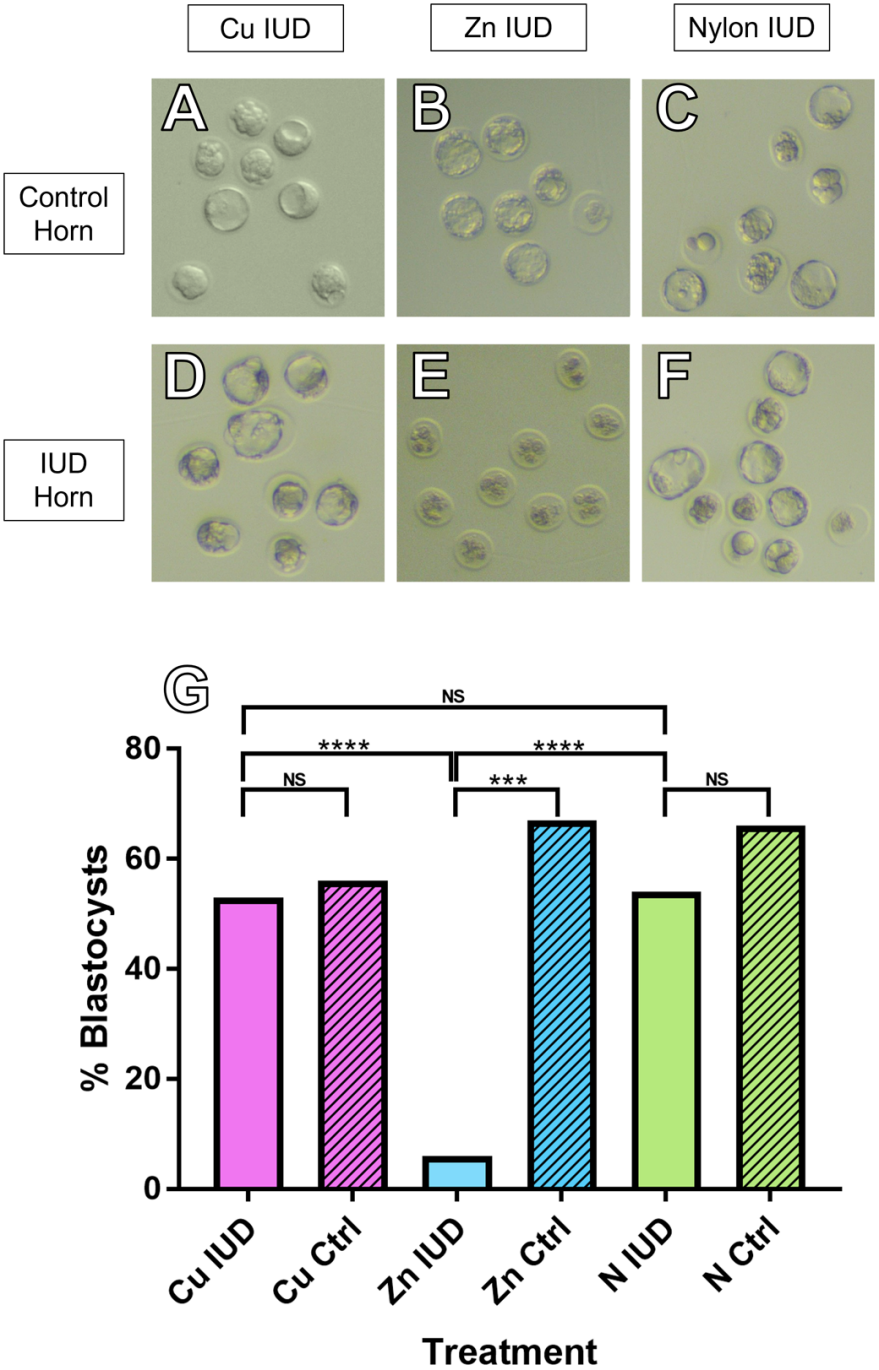
**Percentage of blastocysts that developed from healthy rat embryos extracted from oviducts connected to IUD-treated and control horns at ED2 and cultured until ED5.** (A-F) Representative images of Cu IUD (*n*=25), Cu Ctrl (*n*=21), Zn IUD (*n*=24), Zn Ctrl (*n*=25), N IUD (*n*=14) and N Ctrl (*n*=13) pooled from at least three experiments. (G) Chi-squared analysis with Fisher's exact test was employed, and Bonferroni adjusted *P*-values (*P*<0.008) were used to determine significance for specific comparisons. ***P<0.001 and ****P<0.0001.

To determine whether exposure to a copper or zinc IUD had an impact on preimplantation embryo development, healthy embryos collected from the three rat IUD models on ED2 were cultured until ED5. An adjusted *P*-value of *P*<0.008 was used to determine significance. Significantly fewer embryos from the zinc IUD-treated horns developed into blastocysts compared to those from the control horn (7% versus 68%; *P*<0.0001) and from the nylon IUD horn (7% versus 54%; *P*<0.0001) and copper IUD horn (7% versus 53%; *P*<0.0001) (Fig. 3). The majority of zinc IUD-treated embryos arrested at the two-cell stage. The copper and nylon IUDs did not significantly affect embryo development into blastocysts.

### Zinc impacts early embryo development in a dose-dependent manner

**Fig. 4.**
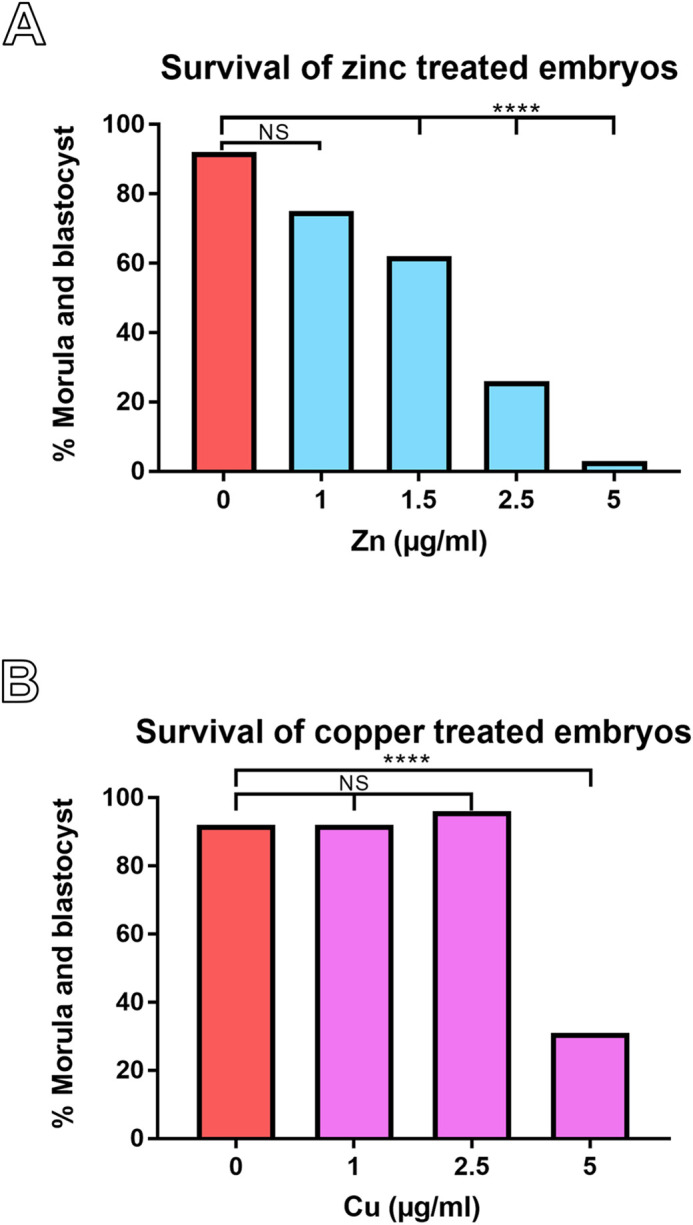
**Survival of mouse embryos cultured in zinc-containing media and copper-containing media from ED1 until ED5, compared to control embryos cultured in normal medium.** Chi-squared analysis with Fisher's exact test was employed, and Bonferroni adjusted *P*-values (A; *P*<0.01, B; *P*<0.0125) were used to determine significance for specific comparisons. (A) Survival of zinc-treated embryos: Zn 1 μg/ml (*n*=28), 1.5 μg/ml (*n*=32), 2.5 μg/ml (*n*=23), 5 μg/ml (*n*=32) and control (*n*=207) pooled from at least three experiments. (B) Survival of copper-treated embryos: Cu 1 μg/ml (*n*=26), 2.5 μg/ml (*n*=31) and 5 μg/ml (*n*=35) pooled from at least three experiments. ****P<0.0001.

Concentrations of zinc and copper required to impact early embryo development were determined by culturing mouse embryos in a range of concentrations from the zygote to blastocyst stage. An adjusted *P*-value of *P*<0.008 was used to determine significance. At a concentration of 1 μg/ml, zinc did not significantly decrease blastocyst development compared to controls (75% versus 92%; *P*=0.0102) (Fig. 4A). Blastocyst development significantly decreased in a dose-dependent manner, compared to controls, when exposed to zinc at a concentration of 1.5 μg/ml (63% versus 92%; *P*<0.0001), 2.5 μg/ml (26% versus 92%; *P*<0.0001), and 5 μg/ml (3% versus 92%; *P*<0.0001) (Fig. 4A). A comparison to the release rate of metallic zinc from the ZnIUD used in the *in vivo* study is provided in Fig. S1. There was no significant difference in blastocyst development, compared to controls, when embryos were exposed to 1 μg/ml (92% versus 92%; *P*>0.9999) and 2.5 μg/ml (97% versus 92%; *P*=0.7061) of copper (Fig. 4B). Blastocyst development significantly decreased when exposed to copper, compared to controls, only at a concentration of 5 μg/ml (31% versus 92%; *P*<0.0001) (Fig. 4B).

### Embryos treated with zinc exhibit decreased attachment rates

**Fig. 5.**
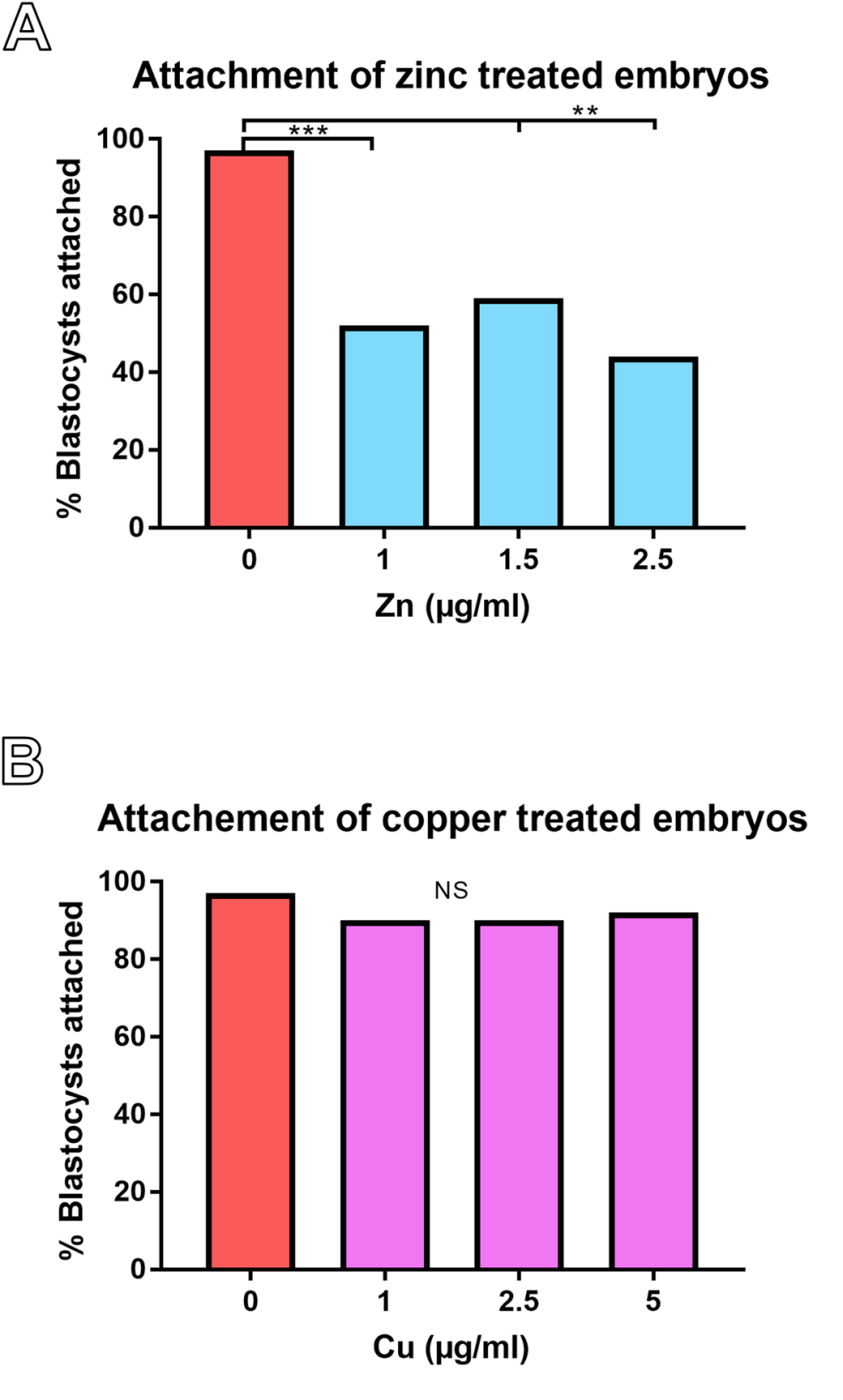
**Attachment of mouse blastocysts treated with zinc and copper to untreated Ishikawa cells.** Chi-squared analysis with Fisher's exact test was employed, and Bonferroni adjusted *P*-values (*P*<0.01) were used to determine significance for specific comparisons. (A) Attachment of zinc-treated embryos: Zn 1 μg/ml (*n*=23), 1.5 μg/ml (*n*=22), 2.5 μg/ml (*n*=9) control (*n*=29) pooled from at least three experiments. (B) Attachment of copper-treated embryos: Cu 1 μg/ml (*n*=21), 2.5 μg/ml (*n*=30), 5 μg/ml (*n*=13) and control (*n*=29) pooled from at least three experiments. **P<0.001 and ***P<0.0001.

After treatment with zinc and copper, surviving mouse blastocysts were placed on untreated Ishikawa cells to determine effects on attachment. An adjusted *P*-value of *P*<0.01 was used to determine significance. All concentrations of zinc resulted in significantly fewer embryos attaching to cells compared to controls, 1 μg/ml (52% versus 97%; *P*<0.0002), 1.5 μg/ml (59% versus 97%; *P*<0.0012) and 2.5 μg/ml (44% versus 97%; *P*<0.0014) (Fig. 5A). Exposure of embryos to copper did not affect blastocyst attachment rates, 1 μg/ml (90% versus 97%; *P*=0.5650), 2.5 μg/ml (90% versus 97%; *P*=0.6120) and 5 μg/ml (92% versus 97%; *P*=0.5285) (Fig. 5B).

### Exposure of Ishikawa cells to zinc or copper does not impact blastocyst attachment

**Fig. 6.**
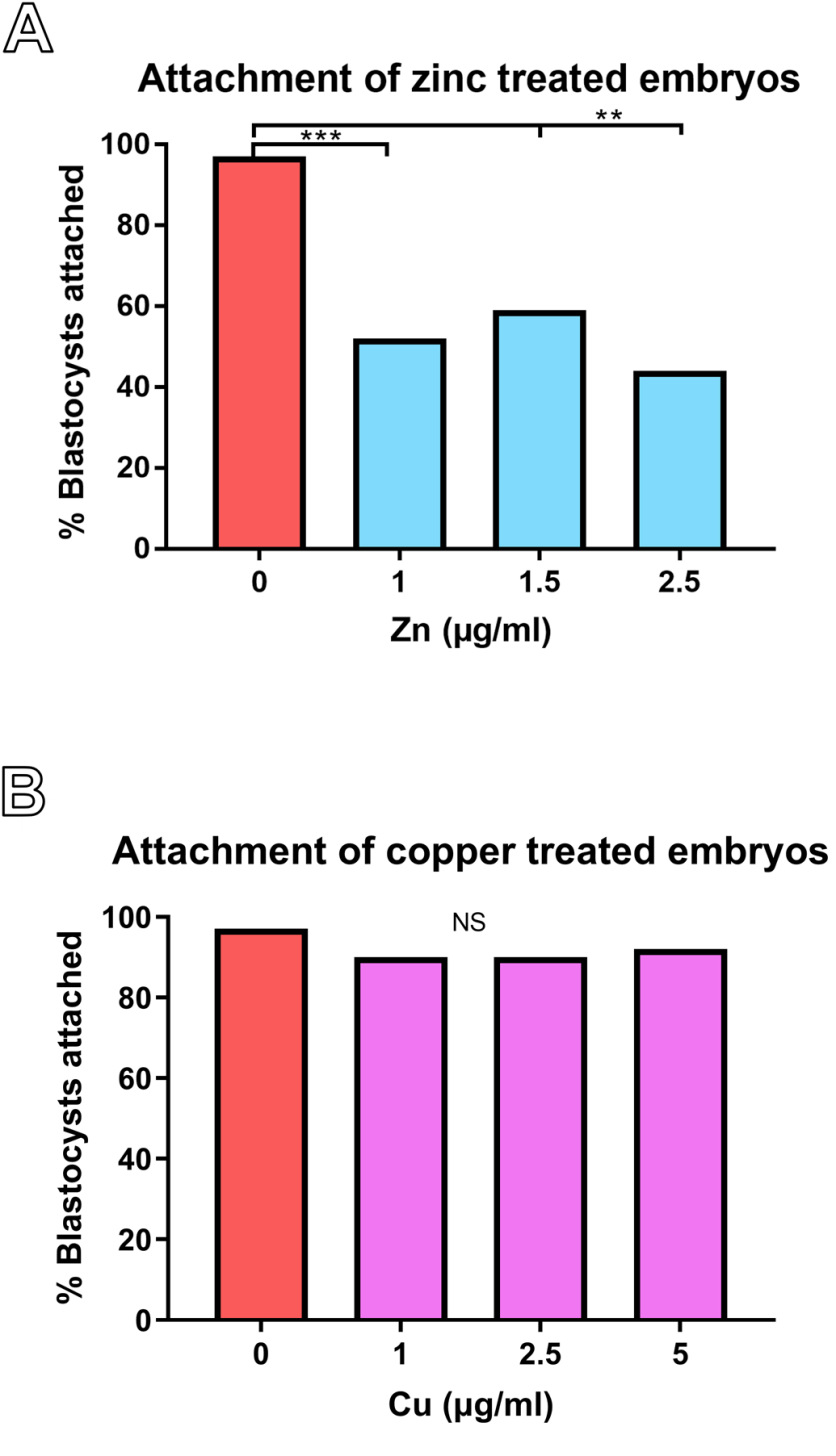
**Attachment of untreated mouse blastocysts to zinc- and copper-treated Ishikawa cells.** Chi-squared analysis with Fisher's exact test was employed, and Bonferroni adjusted *P*-values (*P*<0.01) were used to determine significance for specific comparisons. (A) Attachment of embryos to zinc-treated cells: Zn 1 μg/ml (*n*=27), 1.5 μg/ml (*n*=28), 2.5 μg/ml (*n*=27) and control (*n*=29) pooled from at least three experiments. (B) Attachment of embryos to copper-treated cells: Cu 1 μg/ml (*n*=27), 2.5 μg/ml (*n*=28), 5 μg/ml (*n*=27) and control (*n*=29) pooled from at least three experiments. There was no significant difference between all treatment groups. **P<0.001 and ***P<0.0001.

To examine whether zinc and copper impacted adhesion by affecting the endometrial epithelial cells, cultured cells were treated with zinc and copper, and an attachment assay was performed with untreated mouse embryos. An adjusted *P*-value of *P*<0.01 was used to determine significance. There was no significant difference between any of the treatment groups. There was no significant difference between attachment of blastocysts to control cells and cells treated with zinc at a concentration of 1 μg/ml (100% versus 97%; *P*>0.9999), 1.5 μg/ml (93% versus 97%; *P*=0.6115) and 2.5 μg/ml (85% versus 97%; *P*=0.1855) (Fig. 6A). There was no significant difference between attachment of blastocysts to control cells and cells treated with copper at a concentration of 1 μg/ml (96% versus 97%; *P*>0.9999), 2.5 μg/ml (93% versus 97%; *P*=0.6115) and 5 μg/ml (93 versus 97%; *P*=0.6045) (Fig. 6B).

### Exposure to zinc during the mid-two-cell stage does not prevent mouse blastocyst development

**Fig. 7.**
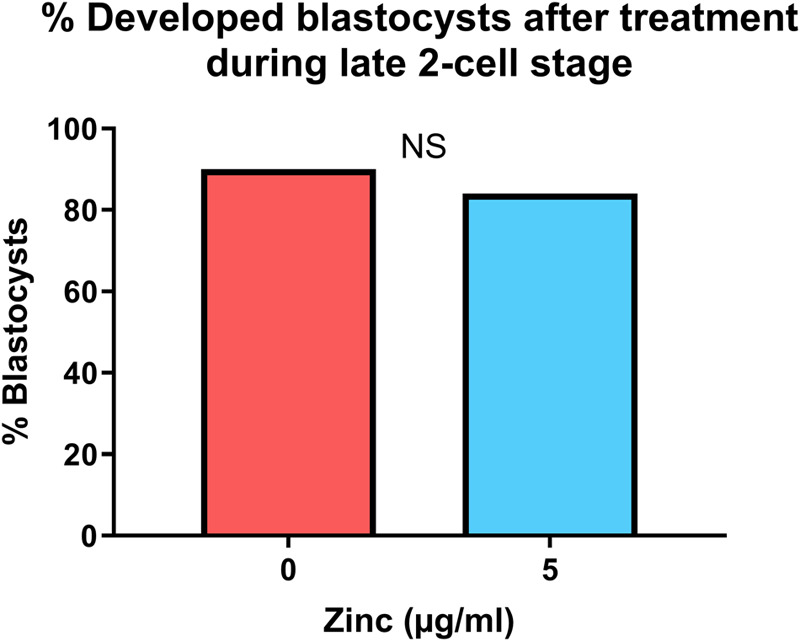
**Development of mouse blastocysts after treatment with zinc during the mid-two-cell stage compared to controls.** Chi-squared analysis with Fisher's exact test was employed. Zn 5 μg/ml (*n*=37) and control (*n*=71) from one experiment. There was no significant difference between treatment groups.

To determine how long the embryo is sensitive to zinc, mouse embryos at the mid-point of the two-cell stage were treated with 5 μg/ml zinc, the concentration previously established to significantly prevent blastocyst development when provided at the zygote/early-two-cell stage (above). There was no significant difference between controls and zinc-treated embryos (90% versus 84%; *P*=0.3617) (Fig. 7).

## DISCUSSION

### Copper and zinc IUDs do not prevent fertilisation

Neither the copper nor the zinc IUD prevented the fertilisation of oocytes in the rat IUD model. Fertilised embryos were recovered from all rat uterine horns containing an IUD, with fertilisation rates of 100% in all treatment groups except the zinc and nylon IUD where one unfertilised oocyte was recovered. This indicates that prevention of fertilisation is not the primary MOA of either IUD material in this model. This is in agreement with an earlier study on the MOA of the copper IUD, which similarly found that although copper IUDs prevented pregnancy in rabbits, they did not inhibit fertilisation, recovering only embryos from the copper-treated horns (Polidoro and Black, 1970). Further studies in rodents have concluded that copper IUDs have a post-fertilisation contraceptive target, by inhibiting embryo implantation (Chang and Tatum, 1970; Kincl and Rudel, 1971; Middleton and Kennedy, 1975). Our work supports this conclusion, as all recovered rat embryos are fertilised and develop into normal blastocysts; yet, in the same model, the copper IUD provides 100% contraceptive efficacy by day 9 of pregnancy, post implantation (Shankie-Williams et al., 2022). Whilst our *in vitro* work also shows that copper may impact development of mouse embryos to the blastocyst stage at high concentrations, it appears that zinc operates at an earlier stage and is more effective at lower concentrations.

### Zinc has a unique target, the early embryo

Embryos recovered from rat uterine horns containing a zinc IUD exhibited disrupted development. On ED2 nearly 50% of embryos from the zinc IUD group exhibited uneven cleavage or degeneration, and, despite culturing in normal media from ED2 to ED5, only one embryo developed into a blastocyst. Thus, this experiment revealed that exposure to the zinc IUD *in vivo* in the first few days of development impacts either gametes or the early embryo such that development to the blastocyst stage is impeded. To further elucidate the timing of the mechanism, embryos were collected at the zygote and mid-two-cell stage from untreated mice and exposed to zinc *in vitro*. Zinc exposure only inhibited blastocyst development when treatment began at the zygote stage. As only treatment initiated after the fusion of the gametes and before the mid-two-cell stage inhibited blastocyst development it is evident that zinc exerts its effect somewhere between the zygote and the very early two-cell embryo.

These findings align with previous work that found that treating two-cell mouse embryos with zinc prevented them all from developing into blastocysts (Andrews et al., 1991; Vidal and Hidalgo, 1993). Although neither study specified the exact time the two-cell embryos were treated, Vidal and Hidalgo (1993) determined that there is a 40% death rate when treating only the zygote, further supporting the hypothesis that the most vulnerable period is the very-early-two-cell stage-embryo. More recently, Yao et al. (2025) found that mouse embryos, exposed to 6 μM ZnCl_2_ (0.4 μg/ml Zn; that of the concentration found in a glass-bottom culture dish) immediately after insemination, exhibited significant growth retardation and chromosomal malformations until the early-two-cell stage. This vulnerability could be due to the metallothionein protein. From the late-one-cell to the early-two-cell embryo the minor and major zygote genome activation occurs, and whilst metallothionein is one of the first genes transcribed, it is not initially metal responsive, only becoming so at the eight-cell stage, when it can be upregulated in response to increased zinc concentrations (Andrews et al., 1991). Before this, the increased exogenous zinc of the IUD could overwhelm the immature metallothionein, leading to embryonic death.

When targeting the two-cell mouse embryo, the minimum concentration of exogenous zinc required to generate a contraceptive effect comparable to that of the copper IUD is 5 μg/ml. However, cultured endometrial epithelial cells treated with 2.5 μg/ml zinc did not show reduced capacity to attach to blastocysts and have previously been found to be viable up to a concentration of 13 μg/ml (Wu et al., 2012). Since these results reflect those seen previously *in vivo* (Shankie-Williams et al., 2022), it is likely that zinc directly impacting early embryonic development is the primary target.

Although 5 μg/ml was the lowest concentration of zinc to prevent almost all mouse blastocyst formation, with only one embryo developing into a blastocyst, lower concentrations of zinc impacted development in a dose-dependent manner. A significant decline in blastocysts began at 1.5 μg/ml zinc and was further reduced at 2.5 μg/ml; however, these effects are not enough to suggest application as a reliable contraceptive method for humans. Previous studies have found a similar effect, showing a significant decline in mouse blastocysts in 50 μM of ZnCl_2_ (3 μg/ml) and 100% lethality in 100 μM (7 μg/ml) (Andrews et al., 1991; Vidal and Hidalgo, 1993); however, the current study more narrowly defined the range required for high contraceptive efficacy.

### Embryo development is not impacted by exposure to copper *in vivo* but is impeded at higher concentrations *in vitro*

Embryos recovered from rat uterine horns containing copper IUDs did not exhibit disrupted development. Embryos collected on ED2 appeared healthy, with normal morphology, and when cultured to ED5 exhibited similar rates of blastocyst development to controls. Mouse embryos collected at the zygote stage (ED1) and exposed to copper *in vitro* to ED5 exhibited no significant impact to development. However, effects could be seen on mouse embryo survival *in vitro* when exposed to 5 μg/ml copper, with survival reduced to 31%. Similarly, deleterious effects of copper have been seen at the one-cell, two-cell and morula stages *in vitro* at a concentration of 6.5 μg/ml (Vidal and Hidalgo, 1993). Together, these studies indicate that exposure to at least 5 μg/ml copper can impede embryo development; however, the reduced survival rates are not reflective of the near 100% contraceptive efficacy of the copper IUD *in vivo*, using the same model (Shankie-Williams et al., 2022) and in general clinical use. As the concentration of copper in the uterine fluid of copper IUD users during ovulation can be below this, as low as 3.9 μg/ml, it is unlikely that embryo toxicity is the primary MOA of the copper IUD (Arancibia et al., 2003). Although copper has been found to be more cytotoxic than zinc up to 13 μg/ml, in the current study, treatment with copper did not impact the capacity for endometrial epithelial cells to attach to mouse blastocysts (Wu et al., 2012). However, this *in vitro* model system is limited and does not perfectly reflect the *in vivo* environment; there is an absence of immune cells and inflammatory response, which have previously been implicated as being central to the contraceptive effect of the copper IUD (Ortiz and Croxatto, 2007; Serfaty and Yaneva, 1988).

This study found that the minimum concentration of zinc required for a strong contraceptive effect is well within a non-toxic range and further specified the embryonic stage that is responsive to elevated zinc, confirming that zinc has a different target to the copper IUD. This is a promising result for the potential use of a zinc intrauterine contraceptive. Future research should focus on elucidating the exact mechanism of action, achieved via live zinc imaging, proteomic assessment and reactive oxygen species staining of zinc-treated embryos. In addition, future work should also focus on the development of a release mechanism capable of a consistent delivery of 5 μg/ml zinc that could provide a new reliable contraceptive without disruption to hormones or the endometrium.

## MATERIALS AND METHODS

### Impact of *in vivo* IUD exposure on blastocyst development

#### Animals and mating

13 nulliparous female Wistar rats were housed in plastic cages under a 12-h light-dark cycle at 21°C. All rats were between 10 and 12 weeks old and weighed 280-300 g. Rats were fed and watered *ad libitum*. All procedures were approved by The University of Sydney Animal Ethics Committee (Protocol 2018/1387) and comply with National Health and Medical Research Council (NHMRC) Australian Code for the Care and Use of Animals for Scientific Purposes 8th Edition, 2013 (updated 2021). The oestrous cycle and mating of the rats were tracked by vaginal smear and cellular composition (Long and Evans, 1922).

#### IUD insertion surgery

Wistar rats were implanted with IUDs made of either copper, zinc or nylon as described previously (Shankie-Williams et al., 2022). Briefly, under deep anaesthesia, the abdomen was opened by the lateral approach, and the right uterine horn was exposed. The zinc and copper IUDs were made from 1 cm length of 99% pure zinc or copper wire, 0.5 mm in diameter, and were finished with a small loop at one end. The IUD was inserted into the uterine lumen through a small incision in the uterine wall, 1 cm from the ovary. All IUDs were secured in place with a nylon suture through the IUD loop and uterine wall. The control nylon IUD consisted of a continuous loop of nylon 1 cm down the uterine lumen and tied outside the uterine wall in the abdominal cavity. The left horn was left untreated, providing an internal control. Rats recovered over a 1-week period and then mated when a pro-oestrus smear was detected. The presence of sperm in a vaginal smear the following morning confirmed mating and indicated ED0. Animals were sacrificed on ED2 to enable embryo collection.

#### Media for rat embryo culture

On ED2, the oviducts were flushed with Hepes-buffered rat embryo culture medium (Hepes R1ECM 246 mOsm/l, pH 7.4) (Miyoshi et al., 1997) containing 1 mg/ml polyvinyl alcohol (PVA; Sigma-Aldrich; St Louis, MO, USA). Embryos were collected and recorded as developing (even cleavage), degenerate (uneven cleavage and fragmentation with no potential for development) or unfertilised oocytes (Table S1). Healthy embryos were then cultured for 72 h, until ED5, at which time they become blastocysts, in rat embryo culture medium (bicarbonate-buffered R1ECM). At ED5, all blastocysts and dead embryos were recorded (Fig. 1; Table S1).

### Defining minimum concentration for zinc anti-fertility effect *in vitro* using mouse embryos

#### Animals and mating

The Quackenbush Swiss (QS) strain of mice was used (Laboratory Animal Services, University of Sydney). QS yield a high number of oocytes, requiring fewer animals, as well as being an outbred strain. Mice were housed under a 12-h light-dark cycle at 21°C. Ovulation induction of female mice (4-10 weeks old) was achieved by intraperitoneal injection of 10 IU pregnant mare serum gonadotrophin (PMSG; Intervet, Sydney, Australia) followed 48 h later by intraperitoneal injection of 10 IU human chorionic gonadotrophin (hCG; Intervet) as previously described (Morris et al., 2020). Superovulated female mice were then paired with a stud male QS mouse (10-30 weeks old) overnight. The presence of a vaginal plug the following day indicated successful mating, and this was ED0. All procedures were approved by the University of Sydney Animal Ethics Committee (Protocol 2020/1828) and comply with NHMRC Australian Code for the Care and Use of Animals for Scientific Purposes 8th Edition, 2013 (updated 2021).

#### Ishikawa cell culture

The Ishikawa cell line was a gift from Professor Chris Murphy, University of Sydney. Ishikawa cells, derived from human endometrial adenocarcinoma, have characteristics of luminal and glandular epithelium, functional steroid receptors (oestrogen, progesterone, and androgen) and apical adhesiveness (Hannan et al., 2010). Passage 12-13 cells were thawed and plated in 24-well plates (Corning, NY, USA) with Dulbecco's modified Eagle's medium (DMEM; GIBCO, Grand Island, NY, USA) containing 10 (v/v) foetal bovine serum (FBS Bovogen Biologicals Pty Ltd, Essendon, VIC, Australia) and 1% penicillin and streptomycin (Invitrogen, Camarillo, CA, USA).

#### Embryo survival and attachment assays

To determine the minimum concentration of zinc required for contraceptive efficacy, one-cell embryos, differentiated from oocytes by the polar body and two pronuclei, were cultured in either control medium (modHTF) (Morris et al., 2020) or modHTF containing 1 μg/ml, 1.5 μg/ml, 2.5 μg/ml or 5 μg/ml Zn or 1 μg/ml, 2.5 μg/ml or 5 μg/ml Cu, using ZnCl_2_ and CuCl_2_, respectively (Sigma-Aldrich). Ishikawa cells that were near confluence were cultured in either control medium or medium containing 1 μg/ml, 1.5 μg/ml or 2.5 μg/ml Zn or 1 μg/ml, 2.5 μg/ml or 5 μg/ml Cu for 2 days prior to receiving blastocysts. Between four and 12 untreated blastocysts were transferred into each well of treated Ishikawa cells and four to 12 treated blastocysts were transferred onto each well of untreated Ishikawa cells (Fig. 1). Treatment groups were performed in duplicate and repeated three times separately. Total sample sizes for all treatment groups are shown in the figure legends. Co-cultures were incubated undisturbed for 48 h then checked for attachment by blowing media with a glass mouth pipette at the blastocysts whilst examining their position under a dissecting microscope (Wild M3 microscope; Leica Microsystems, Wetlar, Germany). Embryos that did not float away were considered to have attached. Data were pooled from three separate experiments.

#### Statistical analysis

All experimental groups were analysed using chi-squared analysis with Fisher's exact test, and statistical significance was determined with Bonferroni adjusted *P*-values.

## Supplementary Material



10.1242/biolopen.062546_sup1 Supplementary information
