# Supplementary material for: The zinc contraceptive effect: targets, timing, and quantitative thresholds
Source: Biol Open. 2026 Jul 3;15(7):bio062546. doi: 10.1242/bio.062546 (PMC13383112; doi:10.1242/bio.062546)
Supplement: Supplementary information [file biolopen-15-062546-s1.pdf]

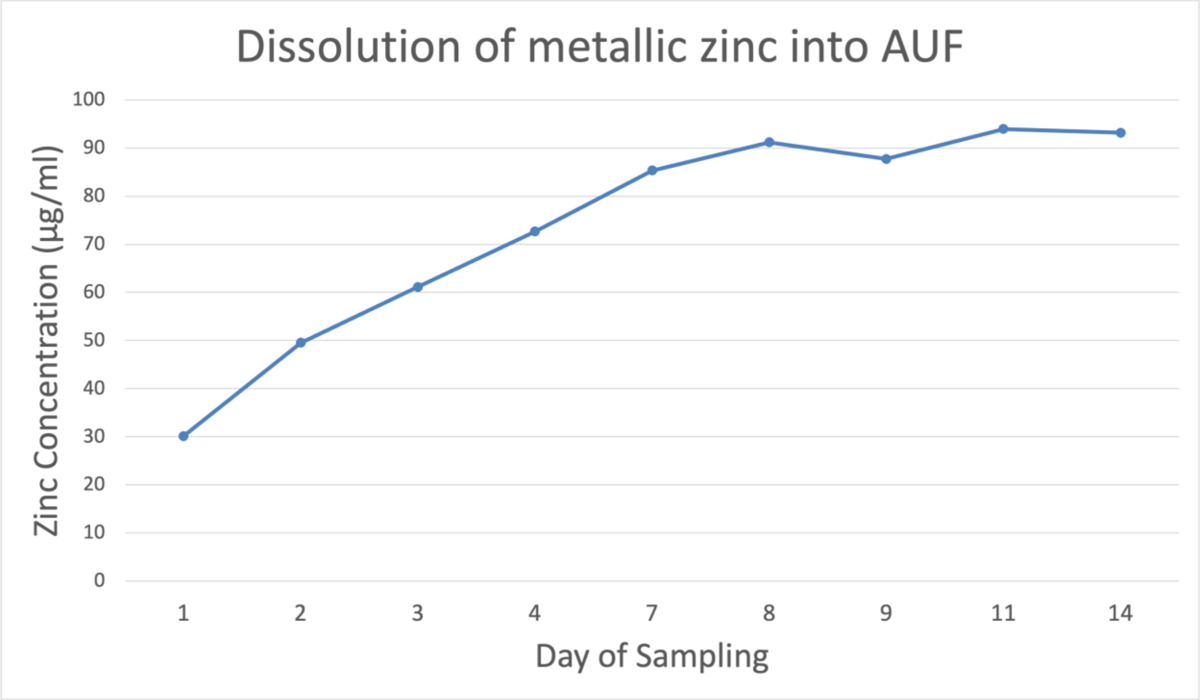

Fig. S1. The dissolution of zinc from metallic zinc IUDs into AUF at 37°C over 14 days. A sample of 20 µl of AUF was taken each day and replaced with 20 µl of fresh AUF. The concentration of zinc in the AUF increased rapidly at first, with the IUD releasing 30 µg/ml in the first 24 h, then gradually slowing until day 7, where it reached 85 µg/ml, and plateaued until day 14 at a concentration of 93 µg/ml.

Table S1. Examples of healthy, degenerating and dead embryos.

Unfertilised	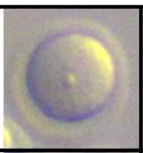	8-cell embryo	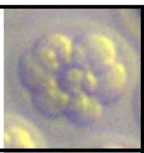	Degenerating embryo	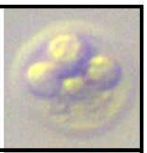
2-cell embryo	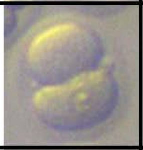	Cavitating blastocyst	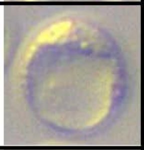	Degenerating embryo	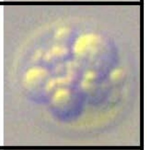
4-cell embryo	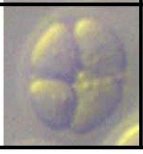	Expanded blastocyst	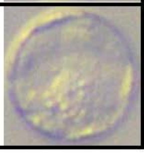	Dead embryo	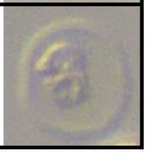

Biology Open • Supplementary information
