# Supplementary material for: The zinc contraceptive effect: targets, timing, and quantitative thresholds
Source: Biol Open. 2026 Jul 3;15(7):bio062546. doi: 10.1242/bio.062546 (PMC13383112; doi:10.1242/bio.062546)
Supplement: Supplementary file 1 [file biolopen-15-062546_review_history.pdf]

The Zinc Contraceptive Effect: Targets, Timing, and Quantitative Thresholds

Margot Day, Samson Dowland and Kirsten Shankie-Williams
10.1242/bio.062546

Editor: Tristan Rodríguez

Review timeline

Original submission:	10 March 2026
Editorial decision:	17 March 2026
First revision received:	6 June 2026
Accepted:	8 June 2026

Original submission

First decision letter

MS ID#: bio.062546

MS Title: The Zinc Contraceptive Effect: Targets, Timing, and Quantitative Thresholds

Authors: Margot Day; Samson Dowland; Kirsten Shankie-Williams

I have now reached a decision on the above manuscript.

The reviewer reports are shown at the bottom of this email.

As you will see, the reviewers gave favourable reports, but raised some critical points that will require amendments to your manuscript. I hope that you will be able to carry these out, because we would like to be able to accept your paper.

At this stage, we also ask you to ensure your manuscript complies with our formatting guidelines – please see our manuscript preparation guidelines for details. Provided you are able to fully address the referees' comments, we are positive about publication of your paper (we accept over 95% of revision submissions) and therefore hope you won't mind any extra work involved in reformatting your manuscript at this point.

Please upload both a 'clean' version of your Word file, along with a highlighted version clearly showing where you have made changes in the revised manuscript. Please avoid using 'Track changes' in Word files as these are lost in PDF conversion.

I should be grateful if you would also provide a point-by-point response detailing how you have dealt with the points raised by the reviewers in the 'Response to Reviewers' box. Please attend to all of the reviewers' comments. If you do not agree with any of their criticisms or suggestions please explain clearly why this is so.

Reviewer 1

Comments for the author

© 2026. Published by The Company of Biologists under the terms of the Creative Commons Attribution License (https://creativecommons.org/licenses/by/4.0/).

In this manuscript, the authors investigated the contraceptive action of zinc and copper in both in vivo and ex vivo settings in rats. The researchers collected embryos on embryonic day 2 (ED2) from uterine horns of rats that had one uterine horn implanted with IUDs made of copper, zinc, or nylon, and assessed their viability. Horns from the same females but without IUDs were used as controls. Subsequently, viable embryos were cultured ex vivo until ED5. The authors concluded that fertilisation was unaffected under any of the experimental conditions, but subsequent embryo development was strongly impaired by the presence of zinc IUDs.

In addition, the researchers tested the influence of different concentrations of zinc and copper on the developmental potential of preimplantation mouse embryos and on the ability of these embryos to attach to untreated Ishikawa cells.

Finally, the authors tested whether exposure of Ishikawa cells to zinc or copper would influence the attachment rate of untreated blastocysts. They concluded that zinc affects the early preimplantation period (up to the early 2 cell stage) in a concentration dependent manner but does not influence embryo attachment.

This manuscript may be of interest to researchers working on early development and may have potential clinical implications. However, before this work can be published, several issues need to be addressed:

1.There are two different abstracts for this manuscript. Which one is the correct version?

2.In Fig. 4, the Zn IUD control horn image shows only one blastocyst among eight embryos. This does not appear representative, given that 68% of embryos from the control horn reportedly developed to the blastocyst stage. Please provide a more representative image.

3.Fig. 5. It would be helpful to include representative images of embryos scored for these experiments. Can the authors comment on the developmental stage at which the zinc treated embryos were arrested or dying?

4.In the section "Embryos treated with zinc exhibited decreased attachment rates"—were mouse or rat embryos used? This needs to be clearly stated in the title or in the first sentence.

5.In the section "Exposure of Ishikawa cells to zinc or copper does not impact blastocyst attachment", the origin of the embryos (mouse or rat) must be provided.

6.For both sections "Embryos treated with zinc exhibited decreased attachment rates" and "Exposure of Ishikawa cells to zinc or copper does not impact blastocyst attachment", the definition of "attachment" should be provided, preferably accompanied by representative images of attached and non attached embryos.

7.In the section "Exposure to zinc during the mid 2 cell stage does not prevent blastocyst development", please provide the origin of the embryos (mouse or rat). In addition, the authors should consider providing representative images of embryos after culture for each group.

8.In the discussion, the authors state: "Our work supports this conclusion, with embryos exposed to copper both in vitro and in vivo developing into normal blastocysts." However, this statement is not fully aligned with their own results, as they observed a significant decrease in mouse embryos reaching the blastocyst stage following exposure to higher concentrations of copper (Fig. 5B).

9.Throughout the discussion, the species used (rat or mouse embryos) should be clearly stated for each treatment discussed.

Reviewer 2

Comments for the author

© 2026. Published by The Company of Biologists under the terms of the Creative Commons Attribution License (https://creativecommons.org/licenses/by/4.0/).

The study from Shankie-Williams et al., addresses the challenge of finding new effective non-hormonal contraception methods with fewer or no side effects. The work presented in this manuscript is a continuation of previous research by the authors which showed zinc IUDs to be an effective contraceptive alternative to copper IUDs in rats. Copper IUDs are currently the most used non-hormonal contraception method but provoke side effects in a large number of women.

In this paper, the authors evaluate zinc's underlying mechanism of action by comparing sperm (via fertilisation), endometrial (via implantation) and embryo (early development) viability using rat and mouse models. Their conclusion points towards embryo development being impaired in the presence of zinc. The data presented shows clearly that zinc's contraceptive effect takes place between the zygote and 2-cell stage, however, the study does not assess the actual mechanism by which zinc inhibits development. Importantly, how does the concentration of zinc in the in vitro media assays compares to the uterine (in vivo) concentration of zinc while the IUD is present? This is an important question to assess whether the experimental design is appropriate and the results can be translated into in vivo models

The main conclusion is supported by the results. The presence of zinc does not stop fertilisation nor impairs implantation, zinc affects early embryo development prior or during the first cell division as shown by embryo survival rates both in rats and mice.

Figures have proper controls, but as discussed by the authors, it is a limitation that copper does not show a phenotype in the in vitro attachment studies and cannot be used as a positive control for the implantation assays.

The experiments are well thought through, but there is an important point missing in how the concentrations of both zinc and copper in vivo (in the uterine environment when the IUD is present) compare to those used in vitro (e.g. Figure 3 v Figure 5).

There is a sufficient number of experimental repeats and multiple embryos have been used to prove the main hypothesis. However, most of the experiments look at binary results, is there fertilisation? are embryos developing?are embryos attaching? The manuscript shows the results in stacked bar plots but there are no images of the mouse experiments (no images beyond Figure 4), how were the quantitative analysis performed in the other Figures?
It will beneficial to see other type of assays, such as investigation of chromosomal abnormalities or testing for ROS species in the embryo, to understand the mechanism beyond confirming the stage at which it takes place.

Major points:

- What is the concentration of zinc in the uterine environment in the presence of the zinc IUD? How does it compare to the in vitro assays? Could the authors measure this?

- Did the authors try to flush embryos from rats at the blastocyst stage, or at late morula stage? Embryos do not recovered if left in basal media from E2, would the degradation be quicker in vivo in continuous present of zinc?

- The authors mention how a potential mechanism to explain the sensitivity of zygotes to zinc could be the lack of the metallothionein protein which prevents oxidative stress from free metal ions. Have they considered staining embryos for ROS species (commercially available) to check this hypothesis?

- Another mechanism discussed in the discussion is chromosomal abnormalities, have the authors considered looking into this in more detail for this or future studies?

- The attachment experiments in Figure 6 are interesting and useful for the study. Can the authors explain how they quantified the results using a diagram or images?

- Can the authors discuss how will they continue the mechanistic research? Proteomics or RNAseq studies will be out of the scope for this piece of work but would be interesting to read how the

© 2026. Published by The Company of Biologists under the terms of the Creative Commons Attribution License (https://creativecommons.org/licenses/by/4.0/).

authors are considering progressing in the understanding of zinc MOA. Could it also be related to the cell division machinery

- There is a section in the discussion mentioning that the mechanism might be affecting either the gametes or the early embryo. Since fertilisation takes place, the authors assume the effect happens between zygote and 2-cell stage, however, the oocyte being affected cannot be completely ruled out as this could be seen with alterations in the maternal proteins needed for the early development. A more in depth characterisation is needed.

Minor comments:

- In the M&M section, "recorded as developing, degenerate or unfertilised oocytes", can the authors add a schematic of how these embryos will look like or pictures?

- Specify animal model in the Figures.

- In Figure 5, the text focuses on embryos reaching the blastocyst stage but the y-axis says: 'morula and blastocyst'. Can the number of embryos reaching either morula or blastocyst be shown separately? What is the rationale for collating both

- The last section of results/Figure 8 "Exposure to zinc during the mid-2-cell stage does not prevent blastocyst development" could be explained in a clearer manner. It is a nice experiment to find the window of action for zinc but the text is not clear about the comparison, only mentioning the 2-cell stage. The text needs rewording to mention the previous experiments using zygotes, albeit briefly.

- Amend title to not overstate findings, the paper clearly shows the stage at which zinc has an effect but not the mechanism.

Reviewer's Responses to Questions

Experimental quality
Does each figure have the proper controls?
If 'No', please indicate reasons in Comments for Author box below.
Reviewer #1:
- Yes

Reviewer #2:
- Yes

Were the data analyzed using appropriate statistical tests?
If 'No', please indicate reasons in Comments for Author box below.
Reviewer #1:
- Yes

Reviewer #2:
- Yes

Reproducibility
Were experiments performed using adequate number of biological replicates?
If 'No', please indicate reasons in Comments for Author box below.
Reviewer #1:
- Yes

Reviewer #2:
- Yes

Does the methods section provide sufficient detail to permit reproducibility?
If 'No', please indicate reasons in Comments for Author box below.
Reviewer #1:
- Yes

Reviewer #2:

© 2026. Published by The Company of Biologists under the terms of the Creative Commons Attribution License (https://creativecommons.org/licenses/by/4.0/).

- No

Completeness
Are the manuscript's conclusions supported by the data?
If 'No', please indicate reasons in Comments for Author box below.
Reviewer #1:
- Yes

Reviewer #2:
- Yes

Scholarship
Do the authors cite and discuss the merits of data that would argue for and against their conclusion?
If 'No', please indicate reasons in Comments for Author box below.
Reviewer #1:
- Yes

Reviewer #2:
- Yes

Does the manuscript title & abstract accurately reflect the contents of the manuscript, without hyperbole?
If 'No', please indicate reasons in Comments for Author box below.
Reviewer #1:
- Yes

Reviewer #2:
- No

First revision

Author response to reviewers' comments

Comments from the Reviewers:

Reviewer 1

1. There are two different abstracts for this manuscript. Which one is the correct version?

Apologies, a prior version that isn't within the word limit was mistakenly copied into the abstract section during submission. The correct abstract is that which was in the body of the manuscript. This has now been rectified and the correct abstract appears in both sections.

2. In Fig. 4, the Zn IUD control horn image shows only one blastocyst among eight embryos. This does not appear representative, given that 68% of embryos from the control horn reportedly developed to the blastocyst stage. Please provide a more representative image.

Thank you for highlighting this, we have updated the figure and replaced this image with a more representative image.

3. Fig. 5. It would be helpful to include representative images of embryos scored for these experiments. Can the authors comment on the developmental stage at which the zinc treated embryos were arrested or dying?

During the study we observed that zinc most often causes arrest at the 2-cell stage, we have now added this information at line 246-247 (highlighted). While we did not capture images of these embryos each day, we have now provided a table of example embryos at different stages of development and degeneration in the supplementary materials, line 658-659.

© 2026. Published by The Company of Biologists under the terms of the Creative Commons Attribution License (https://creativecommons.org/licenses/by/4.0/).

4. In the section "Embryos treated with zinc exhibited decreased attachment rates"—were mouse or rat embryos used? This needs to be clearly stated in the title or in the first sentence.

Thank you for pointing out the need for clarification. This experiment utilised the surviving mouse blastocysts from the previous experiment. This has now been specified in line 301.

5. In the section "Exposure of Ishikawa cells to zinc or copper does not impact blastocyst attachment", the origin of the embryos (mouse or rat) must be provided.

We have clarified line 330 to specify that mouse embryos were used.

6. For both sections "Embryos treated with zinc exhibited decreased attachment rates" and "Exposure of Ishikawa cells to zinc or copper does not impact blastocyst attachment", the definition of "attachment" should be provided, preferably accompanied by representative images of attached and non-attached embryos.

To assess the attachment capability of blastocysts, we used the method previously described by Green, C., J. et al (2014). They are first placed on a confluent layer of Ishikawa cells and left for 48 h to enable attachment. Media is then gently blown across the culture plate and those which remain in place are considered attached and those which move across the cells have not attached. This definition and reference have now been added to the methods section on line 182-186. As this is a dynamic process, unfortunately still images of these embryos do not show any characteristic differences between attached and unattached embryos.

7. In the section "Exposure to zinc during the mid 2 cell stage does not prevent blastocyst development", please provide the origin of the embryos (mouse or rat). In addition, the authors should consider providing representative images of embryos after culture for each group.

We have clarified the animal origin on line 372 and 375. As stated previously, we have now provided a table of example embryos at different stages of development and degeneration in the supplementary materials.

8. In the discussion, the authors state: "Our work supports this conclusion, with embryos exposed to copper both in vitro and in vivo developing into normal blastocysts." However, this statement is not fully aligned with their own results, as they observed a significant decrease in mouse embryos reaching the blastocyst stage following exposure to higher concentrations of copper (Fig. 5B).

Thank you for identifying this, the discussion has been expanded to acknowledge this observation on line 405-412.

9. Throughout the discussion, the species used (rat or mouse embryos) should be clearly stated for each treatment discussed.

We have updated the Discussion to ensure the species of embryo for each treatment being discussed is clearly stated.

Reviewer 2

Major points:

1. What is the concentration of zinc in the uterine environment in the presence of the zinc IUD? How does it compare to the in vitro assays? Could the authors measure this?

This is an important question and something we attempted to determine during the study. However, unfortunately the volume of uterine fluid in a rat is very small and despite multiple attempts, we could not consistently obtain sufficient fluid from the rat uterus to enable elemental analysis. We also tried flushing the uterus with saline, but did not recover the same volume, and

© 2026. Published by The Company of Biologists under the terms of the Creative Commons Attribution License (https://creativecommons.org/licenses/by/4.0/).

without knowing the uterine fluid volume we were adding saline to, this meant we were diluting the uterine fluid by an unknown factor, making it impossible to accurately determine the original concentration of zinc in the uterine fluid. Instead, to enable accurate quantitative analysis we conducted an *in vitro* zinc release study using 1 cm of pure metallic zinc wire (as was used in the *in vivo* model) in 3 ml of artificial rat uterine fluid. On day 1 zinc was at a concentration of 30 ug/ml and on day 7 it was 85 ug/ml. This does not factor in the turnover of uterine fluid and fluctuating volumes *in vivo* but it does suggest that a concentration of at least 5 ug/ml would be expected. This supporting data has been added to supplementary materials.

For the *in vitro* embryo survival assays we were primarily aiming to determine the *minimal* concentration of zinc required for the contraceptive effect, rather than trying to mimic the exact concentration elicited by the IUD *in vivo*. This has been clarified in line 170-171 of the methods section.

2. Did the authors try to flush embryos from rats at the blastocyst stage, or at late morula stage? Embryos do not recovered if left in basal media from E2, would the degradation be quicker in vivo in continuous present of zinc?

No, we didn't try to flush embryos at later stages in this study, but it would be a great addition to future work to elucidate the time of arrest *in vivo*. We expect that longer exposure to zinc *in vivo* would not make much difference as we have identified a distinct window of vulnerability at first cleavage, after which time there is no apparent effect of zinc on blastocyst development (Figure 7).

3. The authors mention how a potential mechanism to explain the sensitivity of zygotes to zinc could be the lack of the metallothionein protein which prevents oxidative stress from free metal ions. Have they considered staining embryos for ROS species (commercially available) to check this hypothesis?

Thank you for the suggestion, this would be another interesting avenue for future work to explore the causal mechanism further. We have now added this to the discussion on line 492.

4. Another mechanism discussed in the discussion is chromosomal abnormalities, have the authors considered looking into this in more detail for this or future studies?

Yes, this is absolutely part of our planned future work to evaluate the safety of such a contraceptive. As we aim for our work to progress towards development of a future contraceptive product, this will be an essential avenue for us to explore/ensure safety.

5. The attachment experiments in Figure 6 are interesting and useful for the study. Can the authors explain how they quantified the results using a diagram or images?

To assess the attachment capability of blastocysts, we used the method previously described by Green, C., J. et al (2014). They are first placed on a confluent layer of Ishikawa cells and left for 48 h to enable attachment. Media is then gently blown across the culture plate and those which remain in place are considered attached and those which move across the cells have not attached. This definition and reference have now been added to the methods section on line 182-186. As this is a dynamic process, unfortunately still images of these embryos do not show any characteristic differences between attached and unattached embryos.

6. Can the authors discuss how will they continue the mechanistic research? Proteomics or RNAseq studies will be out of the scope for this piece of work but would be interesting to read how the authors are considering progressing in the understanding of zinc MOA. Could it also be related to the cell division machinery

Whilst we agree that such downstream analysis is out of scope for this paper, we have started proteomics on IUD-treated uteri and aim to also analyse IUD-treated embryos. In future we will also perform live imaging of zygotes stained with a zinc fluorophore to visualise zinc transport during cleavage in embryos exposed to zinc. This has now been added to line 491 of the Discussion. This current paper provides first confirmation of the reproductive target and the window of this effect.

© 2026. Published by The Company of Biologists under the terms of the Creative Commons Attribution License (https://creativecommons.org/licenses/by/4.0/).

Our subsequent research aims to narrow down the specific mechanistic cell biology that leads to this effect on the embryo during this window of time.

7. There is a section in the discussion mentioning that the mechanism might be affecting either the gametes or the early embryo. Since fertilisation takes place, the authors assume the effect happens between zygote and 2-cell stage, however, the oocyte being affected cannot be completely ruled out as this could be seen with alterations in the maternal proteins needed for the early development. A more in depth characterisation is needed.

This section of the Discussion initially describes the outcome of our *in vivo* experiment in isolation, where zinc IUDs were in place before mating, meaning both gametes and early embryos experienced zinc exposure. It then goes on to outline how our *in vitro* studies refined the target and specific timing by collecting zygotes and 2-cell embryos from untreated mice, meaning these gametes and very early embryos were not exposed to zinc. The outcomes of these *in vitro* experiments indicate that the contraceptive effect is unlikely to be dependent on zinc exposure at these stages (line 423-425).

As the oocytes were not exposed to zinc in these *in vitro* experiments, but zinc treatment still inhibited embryo development, we do not expect any changes to the oocyte to be critical for the contraceptive effect that the present study is focused on. However, we would like to broaden our approach in future work and develop a deeper understanding of how exposure to exogenous zinc may impact the gametes, beyond simply contraception. We aim to conduct proteomics on both sperm and oocytes that are exposed to zinc. We will also perform similar testing on zygotes recovered from uteri both containing a zinc IUD and untreated animals to assess the impact of zinc exposure on the proteome of developing embryos. We also aim to perform donor-surrogate IVF testing with both zinc-exposed and untreated gametes. Together these will enable definitive determination of the impact of zinc exposure on these cells but are beyond the scope of this initial study.

Minor comments:

1. In the M&M section, "recorded as developing, degenerate or unfertilised oocytes", can the authors add a schematic of how these embryos will look like or pictures?

We have now included a table in supplementary materials with examples of embryos at different stages of development or degeneration. We have added further description of these terms for clarity on line 137-138.

2. Specify animal model in the Figures.

Thank you for drawing attention to this. We have updated the flow chart/s (Figure 1) to specify the animal model used for each specific study. We have also updated all Figure legends to specify the animal model to ensure clarity.

3. In Figure 5, the text focuses on embryos reaching the blastocyst stage but the y-axis says: 'morula and blastocyst'. Can the number of embryos reaching either morula or blastocyst be shown separately? What is the rationale for collating both

The embryos were scored for 5 days following fertilisation. Almost all were either dead, blastocysts or hatching, however 3 were healthy morulae. As such, these could not be classified as unviable as they may just be slow to develop. Due to the very low number, we considered the distinction to be relatively unimportant and simply ensured all embryos developing were counted.

4. The last section of results/Figure 8 "Exposure to zinc during the mid-2-cell stage does not prevent blastocyst development" could be explained in a clearer manner. It is a nice experiment to find the window of action for zinc but the text is not clear about the comparison, only mentioning the 2-cell stage. The text needs rewording to mention the previous experiments using zygotes, albeit briefly.

© 2026. Published by The Company of Biologists under the terms of the Creative Commons Attribution License (https://creativecommons.org/licenses/by/4.0/).

Thank you for highlighting this. We have expanded this section of the Discussion to clarify our explanation. We have elaborated on the comparison to highlight the different points in embryo development that the embryos were treated on line 374-378.

5. Amend title to not overstate findings, the paper clearly shows the stage at which zinc has an effect but not the mechanism.

We have changed the title to avoid overstating findings. The new title is ‘The Zinc Contraceptive Effect: Targets, Timing, and Quantitative Thresholds’. The new title is now on line 2-3.

Second decision letter

MS ID#: bio.062546R1

MS Title: The Zinc Contraceptive Effect: Targets, Timing, and Quantitative Thresholds

Authors: Margot Day; Samson Dowland; Kirsten Shankie-Williams

I am happy to tell you that your manuscript has been accepted for publication in Biology Open, pending our standard publication integrity checks. It was accepted on 8th June 2026.

© 2026. Published by The Company of Biologists under the terms of the Creative Commons Attribution License (https://creativecommons.org/licenses/by/4.0/).
